# Prevalence and determinants of intercourse without condoms among migrants and refugees in Morocco, 2021: a cross-sectional study

**DOI:** 10.1038/s41598-022-26953-x

**Published:** 2022-12-28

**Authors:** Touria Essayagh, Meriem Essayagh, Firdaous Essayagh, Mourad Rattal, Germain Bukassa, Hajar Lemriss, El Khansa Mahdaoui, Naoufal Himmouche, Mady Fanta Kouyate, Sanah Essayagh

**Affiliations:** 1 Laboratoire Sciences et Technologies de la Santé, Institut Supérieur des Sciences de la Santé, Hassan First University of Settat, Settat, Morocco; 2 Office National de Sécurité Sanitaire des Produits Alimentaires, Oujda, Morocco; 3 Laboratoire Droit Privé et Enjeux de Développement, Faculté des Sciences Juridiques, Economiques et Sociales, Université Sidi Mohamed Ben Abdellah, Fès, Morocco; 4 Department of Indigenous Services Canada/Government of Canada, Health Surveillance and Assessment Unit, First Nations and Inuit Health, Saskatchewan Region, Regina, SK Canada; 5 Ministère de la Santé et de la Protection Sociale, Direction d’Epidémiologie et de Lutte contre les Maladies, Rabat, Morocco; 6 Institut Supérieur des Sciences de la Santé, Laboratoire Ingénierie Didactique, Entrepreneuriat, Arts et Littératures, Hassan First University of Settat, Settat, Morocco; 7 Unité Investigation et Recherche, Département de Surveillance à l’Agence Nationale de Sécurité Sanitaire, Conakry, Guinea; 8 Laboratoire Agroalimentaire et Santé, Faculté des Sciences et Techniques, Hassan First University of Settat, Settat, Morocco

**Keywords:** Diseases, Medical research, Risk factors

## Abstract

With the world's migratory flow, the risk of infection by the human immunodeficiency virus (HIV) among migrants is increasing. The prevalence of intercourse without condoms with a casual or commercial sex partner, a high-risk sexual behavior for HIV infection, is unknown among migrants. The purpose of this study was to determine the prevalence of intercourse without condoms among migrants and the risk factors associated with not using condoms. In Oujda, we conducted a cross-sectional survey of 416 sexually active migrants. We used a multistage sampling method. Face-to-face interviews were conducted with participants to collect socio-demographic information, disease perception, behavioral habits, sexual behavioral habits, and para-clinical parameters. A multivariate logistical regression analysis identified the risk factors associated with high-risk HIV sexual behaviors. The prevalence of intercourse without condoms with a casual or commercial sex partner was 72.8%, with a median age of 25.0 years, and 212 (69.9%) were males. The prevalence of HIV was 0.2%. Being homeless, having difficulty obtaining condoms, and only having a basic education were all risk factors for these sexual behaviors. Migrants with precarious living conditions are at increased risk of having intercourse without condoms. This group must be prioritized by strengthening public health programs targeting the health of migrants as well as the intervention of thematic non-governmental organizations. Vigilant monitoring of the HIV epidemic, with a focus on vulnerable populations, should be a high priority in Morocco.

## Introduction

The human migratory movements experienced by the world in recent decades are unprecedented. In 2020, international migrants in the world reached 281 million, or 3.6% of the world's population^1^. In 2013, with the new migration policy characterized by respect for the human rights of people on the move, Morocco became not only a transit country to Europe but also a host country for people from West and Central Africa, and Syria, with 86,000 migrants in 2014^2^. Several factors can stimulate migration, such as job search, studies, wars, or fear of persecution. However, everyone has the right to access healthcare regardless of their race, religion, political opinions, or economic or social conditions.

The large influx of migrants, refugees, and asylum seekers has intensified global focus on the health of vulnerable populations and prompted host countries to address their health needs. Migrants, refugees, and asylum seekers are a population at risk of infection by sexually transmitted diseases such as the human immunodeficiency virus (HIV), a disease that presents a redoubtable challenge to life and human dignity. This high risk is attributed to the pre-migration characteristics of HIV-endemic countries of origin, as well as the post-migration characteristics marked by the impact of the migratory route, social and ecological determinants of health, and linguistic, cultural, financial, and legal barriers related to access to preventive services in host countries^3^.

The HIV pandemic takes a heavy toll on human lives, individuals, communities, and health systems. In 2021, globally, an estimated 37.7 million people were living with HIV, 1.5 million were newly infected, and 680,000 died^4^. However, in the African region, for the same year, the number of newly infected people appears at 880,000 and deaths at 460,000, while in the Eastern Mediterranean region, new HIV infections are estimated at 41,000 and deaths at 17,000^5^. A Canadian study of refugee patients revealed an HIV prevalence of 2%^6^. That of Greece, conducted at the Greek-Turkish border among migrants and refugees, showed an HIV prevalence of 0.3%^7^.

Morocco is one of the few countries in the region that has launched a strong response to the HIV epidemic in recent decades^8^. In 2020, According to the most recent epidemiological surveillance data produced by the Ministry of Health's Directorate, the prevalence of HIV in the general population remains low and stable (0.08%), with an epidemic concentration among the major population groups most at risk of infection, including sex workers (1.7%), men who have sex with men (4.1%), people who inject drugs (7.1%), and immigrants (3.0%)^9^.

HIV is an epidemic concentrated in low-income countries and afflicting vulnerable populations. Migrants, refugees, and asylum seekers are the most affected, in addition to sex workers, men and women who exchange sex for money or goods, people in prison, men who have sex with men, and people who inject drugs. Migration can encourage several of these behaviors, pushing the migrant to join one of these groups with unprotected sexual intercourses. The migrant can thus serve as a bridge and facilitate the transmission of HIV to migrant populations and to the population of the host country^10^. In Morocco, to thwart the spread of sexually transmitted diseases and reduce the risks of epidemics linked to HIV, a circular from the Ministry of Health on monitoring the health of illegal migrants was launched in 2003. This circular authorizes access to care for migrants in an irregular administrative situation^11^. In 2010, the World Health Organization (WHO) encouraged the promotion of screening activities among people at risk and the management of positive cases^12^. Anonymous testing and free treatment, regardless of the legal status of migrants, are essential elements in responding to the “Leave No One Behind” policy, a policy refusing exclusion and fighting it^13^.

Integrating combination prevention with migrant, refugee, and asylum seeker populations into the response to HIV is a public health priority. It is a step towards considering migrants as a resource for the socio-economic development of the host countries and not as a burden. Investing in the health of migrants, refugees, and asylum seekers is a future investment in the health of the whole population. Assessing the level of knowledge of migrants, refugees, and asylum seekers about HIV infection and intercourse without condoms is essential to facilitate the formulation of preventive messages targeted at migrants living in Morocco and to end the HIV epidemic. Hence, the objective of our study is to describe the state of knowledge of migrants, asylum seekers, and refugees about HIV infection and to measure the prevalence of intercourse without condoms and its associated risk factors.

## Methods

### Study design and population

Oujda occupies a strategic geographical position in Morocco by overlooking Europe to the north and adjoining the Algerian border to the east, which attracts migrant populations^14^. It is one of the three main regions hosting refugees and asylum seekers^2^.

Between November and December 2021, we conducted a cross-sectional study among the sexually active migrant population that have ties with non-profit associations working to improve the health of migrants or the health of vulnerable people. We based the sampling on two stages. The primary unit brought non-profit associations together. The secondary unit was made up of migrants aged 18 and over, sexually active, present in Oujda on the day of the survey and having agreed to participate in the study. A random draw of the order numbers of the migrants was carried out to determine the secondary unit in each chosen primary unit. This method has accelerated the investigation and reduced costs. A simple random draw was used to select sixteen primary units. Each primary unit was made up of 24 people. We excluded pregnant women from the study.

A migrant has been defined as any person of foreign origin, with or without legal status in Morocco, regardless of their date of entry into the country and the more or less prolonged duration of their stay, or even installation^15^. The legal status of migrants was classified into four categories: (i) regular legal status with legal status in the host country; (ii) irregular legal status without legal status in the host country; (iii) asylum seekers who are seeking safety from persecution or harm in a country other than their own and awaiting a response to their refugee claim; (iv) and refugees who are unable or unwilling to return to their country of origin due to a well-founded fear of persecution on account of their race, religion, nationality, membership of a particular social group or opinion policies^16^. The same questionnaire was used and the same trained investigators were involved to limit the measurement bias. The participant received no remuneration due to the limited budget. Data collection was done in a closed room to ensure confidentiality and anonymity.

**Figure 1:**
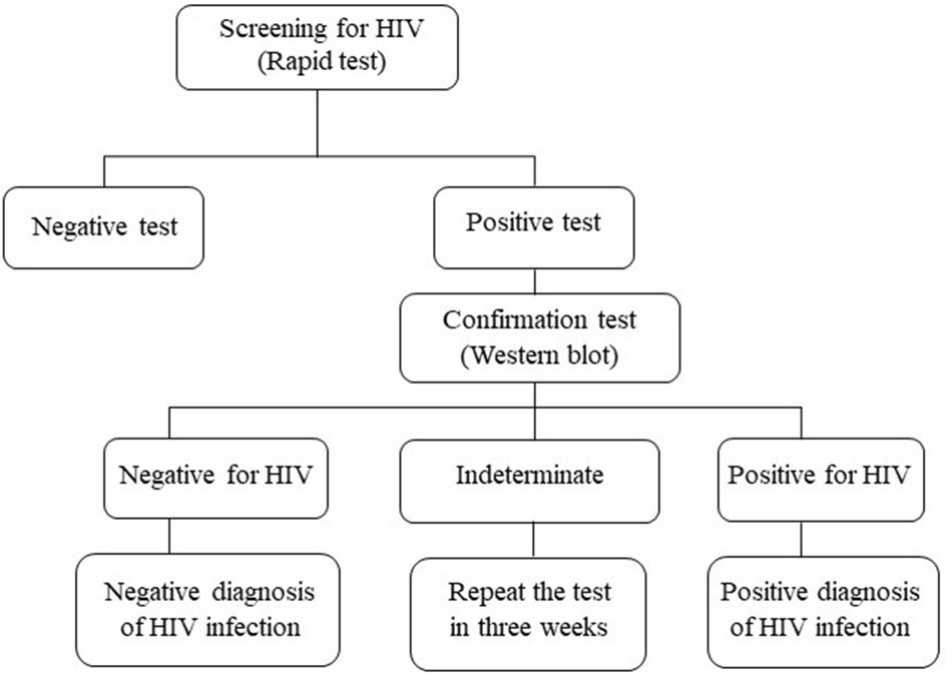
Human immunodeficiency virus diagnostic algorithm in adults, Morocco^17^.

A rapid screening test for HIV used the HIV 1/2/O Tri-line Human Immunodeficiency Virus Rapid Test Device (Abon Biopharm) and/or self-reported HIV and the results were communicated on site. Participants who had positive HIV tests were referred to the referral center for infectious diseases present in Oujda for confirmation by Western blot tests. Confirmed HIV cases were treated. All treatments were free of charge for the participants^17^ (Fig. 1).

### Sample size determination

We set the minimum sample size to 384 migrants based on a 95% confidence interval, a prevalence of high-risk HIV sexual behavior estimates of 50%, and setting the margin of error at 5%.

### Data collection

To collect data in relation to socio-demographic background, perception of the disease by the participant, behavioral habits, sexual behavioral habits, and para-clinical parameters, we used a standardized paper questionnaire during a face-to-face individual interview with the participant. The questionnaire was administered in arabic, french, spanish, or english, which corresponds to the main languages spoken by migrants in Morocco.

### Operational definitions

#### HIV knowledge assessment

We assessed knowledge about HIV through a 17-item questionnaire to which the participant had to answer “yes” or “no”. These items were grouped into three subgroups: eight questions on HIV symptoms, six questions on complications, and three questions on preventive measures. If the participant answered “no” to half of the questions in each subgroup, their knowledge of HIV was said to be unsatisfactory. Conversely, when the participant answered “yes” to half of the questions in each subgroup, he was considered to have “sufficient knowledge” about HIV. The 17 questions were: (i) Is fever a symptom of HIV? (ii) Is asthenia a symptom of HIV? (iii) Are swollen lymph nodes a symptom of HIV? (iv) Is diarrhea a symptom of HIV? (v) Is weight loss a symptom of HIV? (vi) Is oral yeast infection a symptom of HIV? (vii) Is shingles a symptom of HIV? (viii) Is pneumonia a symptom of HIV? Are opportunistic infections complications of HIV? (x) Is wasting syndrome a complication of HIV? (xi) Is lymphoma a complication of HIV? (xii) Is kidney disease a complication of HIV? (xiii) Is liver disease a complication of HIV? (ivx) Are neurological complications a complication of HIV? (xv) Is stopping illicit drug use a preventive measure against HIV? (xvi) Is being careful with piercing and tattooing a preventive measure against HIV? (xvii) Is having safer sex a preventive measure against HIV?

The instrument’s Cronbach’s alpha was 0.86, indicating satisfactory internal consistency for the HIV knowledge assessment questionnaire.

#### Assessment of risk behaviors for HIV infection

The assessment of HIV risk behaviors was carried out by asking questions about the behaviors adopted during the last six months preceding the survey. These behaviors are related to the consumption of alcohol, drug injections, and the fact of having benefited from a piercing or tattoo.

#### Assessment of high-risk HIV sexual behavior

We defined high-risk HIV sexual behavior as any intercourse with a casual or commercial sex partner without condom use. Participants were asked about their sexual identity, age at first sexual intercourse, number of sexual partners, difficulty obtaining condoms, practice of vaginal sex, and practice of anal sex.

### Data management and statistical analysis

The precautionary measures to respect the confidentiality of the information collected and the anonymity of the participants were rigorously respected. The analysis was done with Epi Info version 7.2.0.1. All tests were two-sided and statistical significance was set at a *p*-value of less than 5%. Categorical variables were expressed as numbers and percentages, and continuous variables were expressed as mean and standard deviation. The proportions of categorical variables were compared using the chi-square test or Fisher’s exact test, where applicable. Continuous variables were compared using the analysis of variance test or the Mann–Whitney test, where applicable. Multiple logistic regression was used to identify risk factors associated with high-risk HIV sexual behaviors as defined above, providing odds ratios (OR) and adjusted OR with 95% CI.

### Ethical considerations

The study conformed to the Declaration of Helsinki. The main objective and procedure of the study were given to potential participants. Informed written statement of consent was obtained from all subjects participating in the study. The ethical review board of the Faculty of Medicine and Pharmacy of Rabat reviewed and approved the study protocol.

## Results

### Socio-demographic characteristics

During the study, 416 participants were collected, of whom 303 displayed having intercourse without condoms with a casual or commercial sex partner, which means a prevalence of 72.8%. The median age of the participants was 26.0 years, and 212 (69.9%) were males. Regarding legal status, 184 (44.2%) were in an irregular legal status; 105 (25.2%) were illiterate; and 57 (13.7%) were homeless. The length of stay in Morocco was less than five years in 308 (74.0%) cases (Table 1).

**Table 1:** Socio-demographic and economic characteristics among sexually active migrants, Morocco, 2021.

	Total participants		High-risk HIV sexual behaviors		*p*-value
	n	%	n	%	
Total participants	416	100.0	303	72.8	
Mean age in years ± sd	29.3 ± 11.2		28.0 ± 10.0		< 0.001
Median age in years [IQR]	26.0 [22.0–33.0]		25.0 [21.0–32.0]		0.0002
Age group in years					
18–20	77	18.5	61	79.2	0.01
21–25	119	28.6	95	79.8	
26–30	90	21.6	65	72.2	
31 and more	130	31.3	82	63.1	
Sex					
Male	273	65.6	212	77.7	0.002
Female	143	34.4	91	63.6	
Marital status					
Single^‡^	268	64.4	213	79.5	< 0.001
Partnered^†^	148	35.6	90	60.8	
Education					
Illiterate	105	25.2	59	56.2	0.0001
Elementary	110	26.4	92	83.6	
Middle school	48	11.5	36	75.0	
High school	82	19.7	65	79.3	
College	71	17.1	51	71.8	
Duration of stay in Morocco in years					
< 5	308	74.0	240	77.9	< 0.001
≥ 5	108	26.0	63	58.3	
Legal status					
Irregular	184	44.2	152	82.6	< 0.001
Asylum seeker	154	37.0	113	73.4	
Refugee	33	08.0	5	15.1	
Regular	45	10.8	33	73.3	
Housing type					
Homeless	57	13.7	54	94.7	< 0.001
House*	359	86.3	249	69.4	
Occupation					
No	392	94.2	288	73.5	0.24
Yes	24	05.8	15	62.5	
Monthly income ($)					
> 150	175	42.1	140	80.0	0.005
≤ 150	241	57.9	163	67.6	
Difficulty speaking in the local language					
Yes	278	66.8	207	74.5	0.29
No	138	33.2	96	69.6	
Social support					
No	242	58.2	172	71.0	0.34
Yes	174	41.8	131	75.3	

*sd* standard deviation. *IQR* interquartile range.

*: House means living in house or apartment or reception center.

^†^: Partnered means to be married or to be in concubinage.

^‡^: Single means to be single or divorced or widower.

For qualitative variables, the Pearson chi-2 test estimated the association between the dependent variable and the independent variables when the conditions were valid. For the quantitative variables, we used a comparison test of two means; *p*-value was considered significant when it was less than 0.05.

### Knowledge about HIV infection

Among the 416 participants, 134 (32.2%) had sufficient general knowledge about HIV infection (Table 1). The breakdown by subgroup revealed that 246 (59.1%) were knowledgeable about the symptoms of HIV infection, 331 (79.5%) about its prevention methods, and 147 (35.3%) about its complications.

### Risk behaviors for HIV infection

Analysis of behavioral data showed that 104 (25.0%) had made tattoos or piercings and 81 (19.5%) had consumed alcohol (Table 2).

**Table 2:** Knowledge, risk behaviors and sexual HIV behaviors among sexually active migrants, Morocco, 2021.

	Total participants (n = 416)		High-risk HIV sexual behaviors (n = 303)		*p-*value
	n	%	n	%	
**General knowledge on HIV**					
Insufficient	391	93.9	282	72.1	0.19
Sufficient	25	06.0	21	84.0	
**Variables associated with risk behaviors**					
Consumption of alcohol					
Yes	81	19.5	62	76.5	0.40
No	335	80.5	241	71.9	
Drug user					
Yes	5	01.2	4	80.0	NS
No	411	98.8	299	72.7	
Tattoo or piercing on any part of the body					
Yes	104	25.0	70	67.3	0.14
No	312	75.0	233	74.7	
**Variables associated with sexual behavior**					
Age of first sexual activity in years	17.8 ± 3.2		17.9 ± 3.1		0.45
Number of sexual partners in lifetime					
> 4	158	38.0	122	77.2	0.11
≤ 4	258	62.0	181	70.2	
Vaginal sex					
Yes	416	100.0	303	72.8	NS
No	0	00.0	0	00.0	
Anal sex					
Yes	47	11.3	33	70.2	0.66
No	369	88.7	270	73.2	
Difficulties in obtaining condoms					
Yes	389	93.5	294	75.6	< 0.001
No	27	06.5	9	33.3	
HIV					
Seropositive	1	0.24	1	100.0	0.54
Seronegative	415	99.8	302	72.8	

*sd* standard deviation.

For qualitative variables, the Pearson chi-2 test estimated the association between the dependent variable and the independent variables when the conditions were valid. For the quantitative variables, we used a comparison test of two means; *p*-value was considered significant when it was less than 0.05.

### High-risk HIV sexual behaviors

Among the 416 participants, 303 (72.8%) engaged in intercourse without condoms with a casual or commercial sex partner. The median age was 25.0 years, and 212 (69.9%) were males. Participants with irregular legal status were 152 (50.2%). A total of 59 (19.5%) participants were illiterate, and 54 (17.8%) were homeless (Table 1). The average age at first sexual intercourse was 17.8 ± 3.2 years, and 158 (38.0%) had declared more than four sexual partners during their sexual life. All participants practiced vaginal intercourse, 47 (11.3%) anal intercourse, and 389 (93.5%) had difficulty accessing condoms. One case of HIV was reported (Table 2).

### Bivariate analysis

High-risk HIV sexual behavior, i.e. intercourse without condoms with a casual or commercial sex partner among sexually active migrants was 303 (72.8%). The cut-off *p*-value after the bivariate analysis was set at *p*-value < 0.05. According to the bivariate analysis, we identified eight factors associated with high-risk HIV sexual behavior: (i) male sex (*p* = 0.002); (ii) age 18–20 years (*p *= 0.01); (iii) age 21–25 years (*p* = 0.004); (iv) single (*p* = 0.0001); (v) length of stay in Morocco less than 5 years (*p* = 0.0001); (vi) homeless (*p* = 0.01); (vii) income above $150 (*p* = 0.004); and (viii) difficulties in obtaining condoms (*p* < 0.001) (Table 3).

**Table 3:** Multivariate analysis (odds ratio, *p*-value) of risk factors of high-risk HIV sexual behaviors among sexually active migrants, Morocco, 2021.

	Bivariate analysis			Multivariate analysis complete model		
	COR	95% CI	*p-*value	AOR	95% CI	*p-*value
Sex: Male/female	1.98	[1.27–30.9]	0.002	1.15	[0.66–2.03]	0.60
**Age group in years**						
18–20	2.23	[1.15–4.29]	0.01	0.44	[0.17–1.11]	0.08
21–25	2.31	[1.30–4.10]	0.004	1.02	[0.47–2.24]	0.94
26–30	1.52	[0.85–2.72]	0.15	0.92	[0.43–1.99]	0.84
31–76	1			1		
Marital status: Single/partnered	2.49	[1.60–3.88]	0.0001	1.56	[0.80–3.03]	0.18
Length of stay in Morocco less than 5 years	2.52	[1.57–4.02]	0.0001	1.33	[0.69–2.53]	0.38
Homeless	7.95	[2.43–25.97]	< 0.001	7.01	[1.80–27.29]	0.005
Monthly income more than 150 ($)	1.91	[1.21–3.02]	0.004	2.21	[1.24–3.94]	0.006
**Education**			0.0002			
Illiterate	0.50	[0.26–0.95]	0.03	0.99	[0.40–2.45]	0.99
Elementary	2.00	[0.97–4.12]	0.05	2.94	[1.10–7.89]	0.03
Middle school	1.17	[0.51–2.70]	0.70	1.22	[0.42–3.51]	0.70
High school	1.49	[0.71–3.15]	0.28	1.51	[0.59–3.83]	0.38
College	1			1		
**Legal status**			< 0.001			
Irregular	1.72	[0.80–3.70]	0.16	0.86	[0.32–2.28]	0.76
Asylum seeker	1.00	[0.47–2.12]	0.99	0.67	[0.25–1.80]	0.43
Refugee	0.06	[0.02–0.20]	< 0.001	0.05	[0.01–0.21]	< 0.0001
Regular	1			1		
Difficulties obtaining condoms	6.18	[2.69–14.23]	< 0.001	6.61	[2.56–17.05]	0.0001

*COR* crude odds ratio, *AOR* adjusted odds ratio, *CI* confidence interval.

### Multivariate analysis

After adjusting for the other variables, we identified the following risk factors for high-risk HIV sexual behavior, i.e. intercourse without condoms with a casual or commercial sex partner, among the sexually active migrant population: being homeless.

(AOR = 7.01, 95% CI [1.80–27.29]), having difficulty obtaining condoms (AOR = 6.61, 95% CI [2.56–17.05]), and having an elementary education (AOR = 2.94, 95% CI [1.10–7.89]) (Table 3).

## Discussion

The long latency period of HIV infection in the population makes it difficult to master and control epidemics linked to this disease. Added to this, is the concentrated nature of the epidemic among at-risk groups who are stigmatized and difficult to identify. This stigma reduces access to preventive and curative services. Migrants are among the vulnerable populations at risk for HIV infection. This is a risk exacerbated by a shift in behavior, including sexual behavior, following liberation from relatively rigid societal norms. In our study, the prevalence of migrants who practice intercourse without condoms with a casual or commercial sex partner, a high-sexual risk behavior for HIV infection, was 72.8%. This prevalence was 43% in Kazakhstan in 2015, out of a sample of 422 migrants^18^ and 43% of the 553 Mexican migrants with a recent stay in the border region between Mexico and the United States in 2016 in Tijuana, Mexico^19^.

In a study by Zhuang et al. in 2012 on a sample of 1625 migrants in China, young age was associated with high-risk HIV sexual behaviors^20^. This could be explained by the attitudes that young people have towards sexuality and the fact that young people agree to have sex with casual sex partners or in exchange for money^21^. In our study, age was not associated with high-risk HIV sexual behaviors.

Male gender is known to be a factor consistently associated with sexual behaviors that facilitate the transmission of sexual diseases. These behaviors include multiple sexual partners and casual sex or sex with sex workers^22^. The study of 1219 migrants in Mexico showed that married men who do not live with their spouses were more likely to have sex with a casual partner or a sex worker than their counterparts with their spouse^23^. This could be explained by the perception men have of sex. For these men, they assume they have the right to satisfy their sexual and emotional needs when separated from their spouse^24^. In our study however, male sex and marital status were not associated with practicing intercourse without condoms with a casual or commercial sex partner.While a study conducted on 17,377 migrants in China found no link between a low level of education and high-risk sexual behavior for sexually transmitted diseases^10^, our survey found a significant association. This low level of education could be associated with ignorance about HIV, its mode of transmission, and methods for its prevention.

Insufficient general knowledge about HIV is a risk factor associated with increasing the risk of engaging in intercourse without condoms with casual partners. The study conducted by Pan et in China in 2013 showed that half of migrants believe that HIV can be transmitted by eating with the same utensils as another infected person or by mosquito bites^10^. In their country of origin, talking about sexuality is taboo. Talking about sexual behavior, condom use, and screening for sexually transmitted diseases were not subjects to be discussed with the close entourage of migrants^25^. The literature also reports that migrants who know about prevention methods against sexually transmitted diseases such as HIV adopt safer sexual behaviors ^21^. In our study, insufficient general knowledge about HIV was not a risk factor for having intercourse without condoms with casual partners.

The difficulty of obtaining condoms was associated with high-risk sexual behavior among migrants in Morocco. This may be associated with difficulties obtaining sexual and reproductive health education as well as migrants’ lack of knowledge of places where condoms are distributed for free.

Indeed, a South African study of 943 migrants found that having access to free condoms increased the likelihood of migrants using condoms^26^.

In the current survey, being homeless was listed as a risk factor for intercourse without condoms with casual partners. Indeed, the migratory experience can lead to the absence of a roof, a family, and friends and can engender a feeling of loneliness and a break in social ties to which are added the illegal or legal status, precarious living conditions, absence of work, and barriers to accessing basic services, aggravated by the discrimination of which migrants are victims. All of these conditions lead migrants to seek to compensate for deficits in material and emotional support through sexual behaviors such as intercourse without condoms with sex workers or casual partners^27^. The study by Desgrees du Loû and al. showed that migrants had more unprotected sexual relations during the years when they had no fixed address or irregular legal status^27^. Women, without housing or irregular legal status, were more likely to agree to unwanted sex in exchange for shelter or material support^27^. The precarious living conditions of migrants and the administrative instability in which they find themselves, at least in the first years of their arrival, generate latent anxiety with deleterious effects on their mental health^27^. The symbolic violence imposed by administrative procedures creates instability, insecurity, and situations of discrimination or even harassment. It generates stress and anxieties that affect the health of migrants and expose them to sexuality and the risk of sexually transmitted diseases. The administrative stability marked by a legal status, including refugee status, leads to an increase in self-esteem and a feeling of belonging to the host country, which facilitates their integration and makes them capable of benefiting from basic services, including access to care and prevention methods against sexually transmitted diseases. In our survey, refugee status was a protective factor against intercourse without condoms with casual partners.

## Limitations

Our study had certain limitations, including its cross-sectional nature, which makes it difficult to establish the causal relationship between exposure and the adoption of intercourse without condoms with casual partners. To which is added the prevarication bias encountered during the collection of data in relation to sexual behavior despite the guarantee of anonymity and the use of community actors during data collection. However, our study made it possible to meet its objectives by measuring the prevalence of migrants having intercourse without condoms with casual partners and proposing public health actions to prevent the spread of sexually transmitted diseases, including HIV.

## Conclusion

The results of this research have important implications for maintaining and improving the health of populations in Morocco, especially with regard to human dynamics. After more than 30 years of experience in the fight against sexually transmitted diseases in Morocco, research on HIV among migrants is quasi-absent. The results of this survey show that the homeless, the difficulties of obtaining condoms, and the elementary education level are factors associated with high sexual behavior for HIV. Following these results, it is important to emphasize the need to increase HIV prevention among migrants by taking action on the social and ecological determinants by providing them with shelter and suitable living conditions. In addition, the establishment of community prevention programs against HIV would encourage migrants to adopt healthier and safer sexual behaviors, which would ensure their support for change.

It is important to make migrants aware of the importance of preventing HIV infection through the use of condoms as well as pre-exposure or post-exposure prophylaxis.

## Data Availability

All data generated or analyzed during this study are included in this published article.
